# Mechanistic Study on the Inhibitory Effect of Dandelion Extract on Breast Cancer Cell Proliferation and Its Induction of Apoptosis

**DOI:** 10.3390/biology14080910

**Published:** 2025-07-22

**Authors:** Weifeng Mou, Ping Zhang, Yu Cui, Doudou Yang, Guanjie Zhao, Haijun Xu, Dandan Zhang, Yinku Liang

**Affiliations:** 1 Shaanxi Provincial Key Laboratory of Resource Biology, Provincial-Ministry Joint State Key Laboratory of Qinba Biological Resources and Ecological Environment, Collaborative Innovation Center for Comprehensive Development of Biological Resources in Qinba Mountain Area of Southern Shaanxi, School of Biological Sciences and Engineering, Shaanxi University of Technology, Hanzhong 723000, China; 2 Shaanxi Sanbafule Sci&Tech Co., Ltd., Xi’an 710000, China

**Keywords:** dandelion extract, MDA-MB-231 breast cancer cells, cell cycle, apoptosis, label-free proteomics

## Abstract

This study aimed to investigate the underlying mechanisms by which dandelion extract inhibits the proliferation of breast cancer MDA-MB-231 cells. Dandelion root and leaf extracts were prepared using a heat reflux method and subjected to solvent gradient extraction to obtain fractions with different polarities. MTT assays revealed that the ethyl acetate fraction exhibited the strongest inhibitory effect on cell proliferation. LC-MS analysis identified 12 potential active compounds, including sesquiterpenes such as Isoalantolactone and Artemisinin, which showed significantly lower toxicity toward normal mammary epithelial MCF-10A cells compared to tumor cells (*p* < 0.01). Mechanistic studies demonstrated that the extract induced apoptosis in a dose-dependent manner, with an apoptosis rate as high as 85.04%, and significantly arrested the cell cycle at the S and G2/M phases. Label-free quantitative proteomics identified 137 differentially expressed proteins (|FC| > 2, *p* < 0.05). GO enrichment analysis indicated that these proteins were mainly involved in cell cycle regulation and apoptosis. KEGG pathway analysis revealed that the antitumor effects were primarily mediated through the regulation of PI3K-Akt (hsa04151), JAK-STAT (hsa04630), and PPAR (hsa03320) signaling pathways. Moreover, differential proteins such as PI3K, AKT1S1, SIRT6, JAK1, SCD, STAT3, CASP8, STAT2, STAT6, and PAK1 showed strong correlation with the core components of the EA-2 fraction of dandelion. Molecular docking results demonstrated that these active compounds exhibited strong binding affinities with key target proteins such as PI3K and JAK1 (binding energy < −5.0 kcal/mol). This study elucidates the multi-target, multi-pathway synergistic mechanisms by which dandelion extract inhibits breast cancer, providing a theoretical basis for the development of novel antitumor agents.

## Simple Summary

This study explored how dandelion extract can help fight breast cancer. We found that a specific part of the extract, obtained using ethyl acetate, was most effective at stopping the growth of breast cancer cells (MDA-MB-231) while being less harmful to normal cells. The extract worked by causing cancer cells to die and blocking their growth cycle. Advanced analysis showed that the extract affects important proteins and signaling pathways related to cancer development, such as PI3K-Akt, JAK-STAT, and PPAR. Some of the natural compounds in the extract, like Isoalantolactone and Artemisinin, showed strong potential to bind with key cancer-related proteins. These findings suggest that dandelion extract may serve as a promising source for developing new anticancer treatments.

## 1. Introduction

Breast cancer is one of the most common cancers among women worldwide, with its incidence steadily increasing since the 20th century. In 2020, there were more than 2.2 million new breast cancer cases globally, accounting for 11.7% of all cancer cases in women [1]. China has become one of the countries with the fastest-growing incidence of breast cancer, with approximately 400,000 new cases reported annually [2,3]. Currently, the main treatments for breast cancer include surgery, radiotherapy, chemotherapy, and targeted endocrine/molecular therapy [4]. Although these approaches can significantly reduce tumor burden, their side effects—such as immunosuppression, hair loss, and nausea—often impact patients’ quality of life. Moreover, resistance and toxicity have emerged as major obstacles to chemotherapy [5]. Triple-negative breast cancer (TNBC) is a highly aggressive subtype of breast cancer that lacks expression of estrogen receptor (ER), progesterone receptor (PR), and human epidermal growth factor receptor 2 (HER2), and is thus associated with higher invasiveness, metastasis, and poor prognosis [6]. Therefore, identifying new therapeutic strategies is particularly crucial for TNBC patients. Numerous studies have shown that bioactive compounds extracted from various traditional medicinal plants have attracted widespread attention as important sources of anticancer drugs [7].

Many natural compounds play important roles in cancer treatment by inhibiting cancer cell proliferation and inducing apoptosis. For instance, phytochemicals such as resveratrol and quercetin suppress cancer cell proliferation and promote apoptosis by regulating cell cycle and apoptosis-related pathways [8]. Cell proliferation is governed by multiple signaling pathways, particularly those involving cell cycle regulatory proteins, cyclin-dependent kinases (CDKs), and inhibitory factors such as p21 and p27 [9]. On the other hand, apoptosis, as a type I programmed cell death (PCD), is also modulated by cell cycle-associated proteins. Numerous studies have demonstrated that certain compounds can inhibit cancer cell proliferation by blocking cyclins and their associated kinases, while also enhancing apoptosis by regulating apoptosis-related proteins such as p53, the Bcl-2 family, and caspases [10,11,12]. Therefore, natural phytochemicals can simultaneously suppress tumor cell proliferation and accelerate cancer cell death through apoptosis, showcasing their tremendous potential as promising anticancer agents.

Dandelion (*Taraxacum officinale*) has a sweet-bitter taste and a cold nature, and it enters the liver and stomach meridians. It is rich in sterols, flavonoids, phenolic acids, and volatile oils. It exhibits significant antioxidant, anti-inflammatory, antibacterial, and antitumor activities [13]. However, current research on the use of dandelion in the treatment of female breast hyperplasia and breast cancer is limited. Most studies have focused on crude extracts, and the specific active components responsible for the therapeutic effects remain unclear. Moreover, issues such as low concentrations of effective constituents, poor efficacy, and unclear mechanisms of action still exist. Therefore, this study focuses on screening the active components in dandelion leaves and roots using breast cancer cells as the research model, aiming to identify clearly defined anti-breast cancer compounds and preliminarily explore their mechanisms of action. This work is intended to provide a theoretical basis for the clinical treatment of breast cancer and lay the foundation for the development and application of related natural medicines in the future.

## 2. Materials and Methods

### 2.1. Plant Materials

Dandelion was purchased from the Hanzhong Chinese Herbal Medicine Market in Shaanxi Province.

### 2.2. Chemicals and Reagents

MDA-MB-231 and MCF-10A cell lines (Sangon Biotech Co., Ltd., Shanghai, China); fetal bovine serum (FBS, Cat# 11011–8611, Zhejiang Tianhang Biotechnology Co., Ltd., Hangzhou, China); trypsin cell dissociation solution (Cat# 25200–056,Thermo Fisher Scientific China Co., Ltd., Shanghai, China); penicillin-streptomycin solution 100× (Cat# C0222, Beyotime Biotechnology Co., Ltd., Shanghai, China); MTT, 96-well culture plates, T25 cell culture flasks, and cryogenic vials (Sangon Biotech Co., Ltd., Shanghai, China); Hoechst 33342 dye (Solarbio Life Sciences, Beijing, China); absolute ethanol and ethyl acetate (analytical grade, Tianjin Fuyu Fine Chemical Co., Ltd., Tianjin, China); complete growth medium for MCF-10A human mammary epithelial cells (Cat# M171500, Thermo Fisher Scientific Co., Ltd., Shanghai, China); Annexin V-FITC apoptosis detection kit (Beyotime Biotechnology Co., Ltd., Shanghai, China). All solvents used for chemical analysis were of analytical grade.

### 2.3. Preparation of Crude Extracts from Dandelion Roots and Leaves

A total of 30 g each of dandelion root and leaves were pulverized and passed through a No. 2 sieve. The powdered materials were separately extracted twice by heat reflux using either water or anhydrous ethanol, each lasting 2 h. The extracts were filtered, and the filtrates were combined. After concentration under reduced pressure, aqueous and ethanolic extracts of dandelion roots and leaves were obtained in the form of thick extracts.

### 2.4. Isolation of Ethanol Extract from Dandelion Root

The ethanol extract of dandelion root was concentrated by rotary evaporation to obtain a crude extract. The extract was dispersed and dissolved in an appropriate amount of water, followed by extraction with ethyl acetate five times until the supernatant showed no significant turbidity, yielding an ethyl acetate fraction and an aqueous fraction. Both fractions were filtered, and the solvents were removed to obtain the ethyl acetate extract and aqueous extract, respectively. Subsequently, the components were analyzed using the AKTA pure 25 system with Sephadex G-10 as the stationary phase.

### 2.5. LC-MS Analysis

The dandelion extracts were subjected to non-targeted analysis using LC-MS, and data processing was performed with Compound Discoverer™ 3.0 software, providing information such as predicted chemical names, chemical formulas, molecular weights, retention times, maximum chromatographic peak areas, and mzVault best match scores.

Chromatographic separation was performed using a Waters ultra-performance liquid chromatography (UPLC) system (AcQuity UPLC, Waters Corporation, Milford, MA, USA). Based on the chemical properties of the compounds, a Waters HSS T3 column (100 mm × 2.1 mm, 1.8 μm) was used. The injection volume was 2 μL, the column temperature was maintained at 40 °C, and the flow rate was set at 0.3 mL/min. The mobile phase consisted of solvent A (0.1% formic acid in water) and solvent B (0.1% formic acid in acetonitrile/isopropanol). The gradient elution program was as follows: 0–2 min, 90% A; 2–6 min, 90–40% A; 6–15 min, 40% A; 15.1–17 min, 10% A.

Mass spectrometry was detected using a Q Exactive high-resolution mass spectrometer (Thermo Fisher Scientific, Waltham, MA, USA). Samples were analyzed in both positive and negative ionization modes using electrospray ionization (ESI) under selected ion monitoring (SIM) mode. Optimized MS parameters were as follows: sheath gas flow rate, 40 arb; auxiliary gas flow rate, 10 arb; spray voltage, +3000 V; capillary temperature, 350 °C; and ion transfer tube temperature, 320 °C.

Q Exactive 2.9 software was used for mass spectrometer control. Xcalibur 4.1 was used for data acquisition. Chromatographic peak extraction, alignment, and compound identification were performed using Compound Discoverer 3.0 software. The MS/MS fragmentation and secondary structure analysis were conducted using MS Frontier 7.0. All software packages were obtained from Thermo Fisher Scientific (USA).

### 2.6. Cell Culture of MDA-MB-231

MDA-MB-231 cells were cultured at 37 °C under a CO_2_-free atmosphere in a medium of L-15 supplemented with 10% fetal bovine serum and 1% penicillin-streptomycin. MCF-10A cells were cultured at 37 °C in a humidified atmosphere containing 5% CO_2_ in a complete medium consisting of DMEM/F12 (1:1) supplemented with 5% horse serum, 10 µg/mL insulin, 20 ng/mL epidermal growth factor, 100 ng/mL cholera toxin, and 0.5 µg/mL hydrocortisone.

### 2.7. MTT Assay for Cell Viability

MDA-MB-231 cells in the logarithmic growth phase were harvested by trypsinization, collected, and counted to prepare a cell suspension. The cell density was adjusted to 0.5–1 × 10^5^ cells/mL, and 100 µL of the suspension was seeded into each well of a 96-well culture plate. The cells were incubated overnight at 37 °C in a 5% CO_2_ incubator to allow for adherence, followed by treatment with dandelion extracts. Each well received 100 µL of the extract solution, and cells were incubated for 24 h. Each treatment group included six replicate wells, with a blank group (medium only) and a control group (cells with medium) set up simultaneously.

After incubation, the culture medium was removed, and the wells were washed once with sterile PBS. Then, 200 µL of serum-free MEM medium and 10 µL of 0.5% MTT solution were added to each well. The plates were incubated for 4 h at 37 °C. After incubation, the supernatant was discarded, and 150 µL of DMSO was added to each well. The plates were gently shaken for 10 min to dissolve the formazan crystals completely. Absorbance was measured at 490 nm using a microplate reader. Cell viability and the half-maximal inhibitory concentration (IC50) were subsequently calculated.

### 2.8. Hoechst Staining

Hoechst staining was performed to examine nuclear fragmentation and apoptotic cell morphology. After treatment, cells were fixed with 4% paraformaldehyde for 30 min and stained with Hoechst 33,342 fluorescent dye for 10 min. Following staining, cells were washed three times with sterile PBS and observed under an Olympus IX71 inverted fluorescence microscope (Olympus Corporation, Tokyo, Japan).

### 2.9. Detection of Apoptosis and Cell Cycle in MDA-MB-231 Cells by Annexin V/PI Dual Staining

Flow cytometry was used to detect the levels of apoptosis in cells subjected to different treatments. Briefly, cells were harvested by trypsinization and centrifuged at 300× *g* for 5 min at 4 °C. The cell pellets were washed twice with pre-cooled PBS, centrifuged, and resuspended in a binding buffer. The apoptotic staining solution was added, and the cells were incubated at room temperature in the dark for 15 min. After incubation, the cells were rewashed with binding buffer, resuspended, and analyzed by flow cytometry. Data were collected and analyzed using FlowJo V10.0 software to determine apoptosis and necrosis rates.

For cell cycle analysis, treated cells were washed twice with pre-cooled PBS, digested with trypsin, and collected by centrifugation. After washing with PBS, cells were resuspended and incubated with 2 µL of RNase A at 37 °C in a water bath for 30 min. Subsequently, 50 µL of propidium iodide (PI) staining solution was added, and the cells were incubated at room temperature in the dark for 20 min. Cell cycle distribution was then analyzed using flow cytometry, and the data were processed with ModFit 5.0 software.

### 2.10. Preparation of Samples for Proteomic Analysis

Cells in the logarithmic growth phase were seeded into culture dishes at a density of 2–3 × 10^6^ cells per dish. After normal incubation until cell confluence reached approximately 80%, the old medium was discarded, and the cells were washed once with PBS. Then, 10 mL of medium containing 2 mg/mL EA-2 was added to each dish for the experimental group, while a blank control group was established in parallel. Each group included three biological replicates. The cells were incubated for 24 h in a humidified incubator.

Following incubation, the medium was removed, and adherent cells were digested with trypsin. The digested cells and residual medium were collected into centrifuge tubes and centrifuged at 1000× *g* for 5 min to harvest the cells. The cell pellets were resuspended in PBS and counted, ensuring each group contained 1–2 × 10^7^ cells. Cells were centrifuged again at 1000× *g* for 5 min, and the resulting cell pellets were rapidly frozen in liquid nitrogen and subsequently stored at −80 °C for further proteomic analysis.

### 2.11. Protein Identification and Quantification

Proteomic analysis was performed using an aQExactive mass spectrometer (Thermo Fisher Scientific, Waltham, MA, USA) for label-free quantitative proteomics analysis. Protein identification and quantification were carried out by searching the raw mass spectrometry data against the relevant protein annotation database using MaxQuant 1.5.3.17 software. The protein quantification algorithm used was LFQ (Label-Free Quantification). The specific parameters used for database searching and quantification in MaxQuant are summarized in Supplementary Table S1. Detailed steps of the analysis are provided in Supplementary Section S1.

### 2.12. Molecular Docking

The ligand–protein docking studies were conducted with CB-Dock2 [14]. Compound structures were acquired from PubChem in SDF format and energy-minimized. Protein structures were retrieved from RCSB PDB, hydrogenated, and dehydrated. CB-Dock2 computed the binding affinities (kcal/mol), with the most favorable docking poses selected based on energy scores.

### 2.13. Data Analysis

Data were analyzed using Excel 2016, GraphPad Prism 9, and FlowJo V10 software. Experimental data are presented as means ± standard deviation ($\bar{x}$ ± S) from three independent replicates.

## 3. Results

### 3.1. Effect of Dandelion Extract on the Viability of Human Breast Cancer MDA-MB-231 Cells

**Figure 1:**
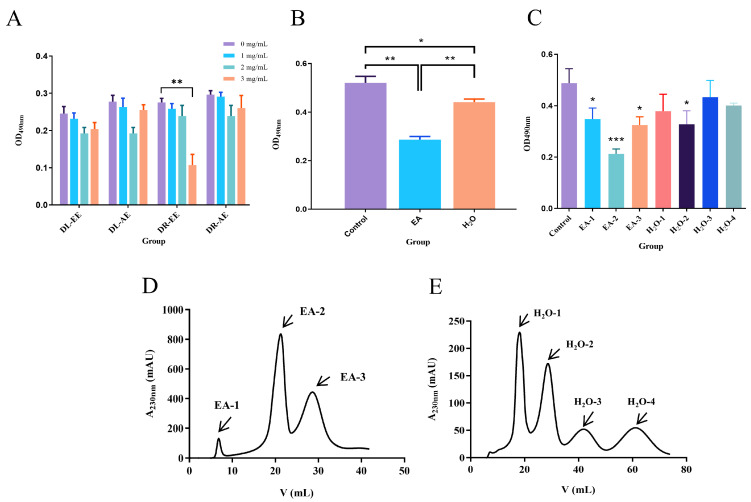
Effects of dandelion extracts on the viability of MDA-MB-231 Cells. (**A**). Screening of the effects of different dandelion root and leaf extracts on MDA-MB-231 cell viability. (**B**). Comparative analysis of the EA and aqueous (H_2_O) fractions of dandelion root on MDA-MB-231 cell viability. (**C**). Inhibitory effects of different fractions on MDA-MB-231 cell viability. (**D**). Chromatogram of the EA fraction from dandelion root. (**E**). Chromatogram of the H_2_O fraction from dandelion root. (Data are presented as the mean ± SD (*n* = 3), and statistical significance was assessed by one-way ANOVA. * *p* < 0.05, ** *p* < 0.01, *** *p* < 0.001 vs. control group).

Dandelion extracts were categorized into four types: dandelion root aqueous extract (DR-AE), dandelion root ethanol extract (DR-EE), dandelion leaf aqueous extract (DL-AE), and dandelion leaf ethanol extract (DL-EE). These four extracts were applied to MDA-MB-231 cells at concentrations of 0, 1, 2, and 3 mg/mL for 24 h, and cell viability was measured by OD at 490 nm using a microplate reader. Among the four extracts, only DR-EE showed significant inhibition of MDA-MB-231 cell viability at 3 mg/mL (*p* < 0.01), indicating that the anti-breast cancer activity of dandelion is primarily concentrated in the root, with DR-EE exhibiting a significant inhibitory effect at this concentration (Figure 1A). Based on this result, DR-EE was selected for further investigation. The extract was partitioned using ethyl acetate, and after solvent removal, two fractions were obtained: the ethyl acetate (EA) fraction and the aqueous (H_2_O) fraction. To assess their biological activity, both fractions were applied to MDA-MB-231 cells, and OD490 nm values were measured. Compared with the control group, both the EA and H_2_O fractions showed significant inhibitory effects on cell proliferation (*p* < 0.05, *p* < 0.01), with the EA fraction exhibiting a more pronounced effect (*p* < 0.01) (Figure 1B).

Subsequently, the EA and H_2_O fractions were separated using Sephadex G-10 gel chromatography, and the eluates were monitored at 230 nm using an AKTA pure 25 system (Figure 1D,E). Seven major fractions were collected and applied to MDA-MB-231 cells. Among them, EA-1, EA-2, EA-3, and H_2_O-2 significantly inhibited cell proliferation compared to the control (*p* < 0.05), with EA-2 (the second subfraction of the ethyl acetate extract) exhibiting the most significant inhibitory effect (*p* < 0.001) (Figure 1C). Therefore, the ethyl acetate fraction was identified as having potent inhibitory activity on human breast cancer MDA-MB-231 cells, and the most effective fraction, EA-2, was selected for subsequent analyses.

### 3.2. Identification of EA-2 Compound Components

**Figure 2:**
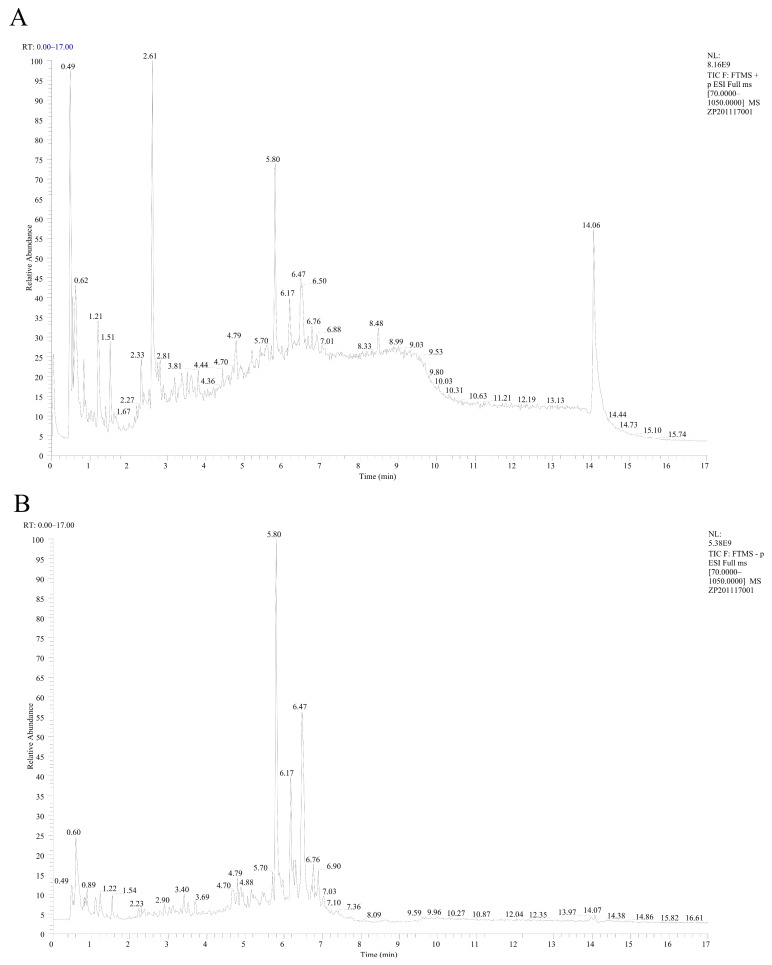
Total ion chromatogram (TIC) of EA-2 in positive ion mode (**A**) and negative ion mode (**B**) from LC-MS analysis.

High-performance liquid chromatography–tandem mass spectrometry (LC-MS) was used to analyze the EA-2 sample, and the total ion chromatogram and mass spectrometry data were obtained (Figure 2A,B). The analysis identified 12 major chemical components in the sample, including Betaine, (R)-Mandelic acid, Azelaic acid, Arglabin, and others. The corresponding MS/MS spectra for each element are shown in Supplementary Figure S1. As shown in Table 1, most of these components exhibit anti-inflammatory activity and may represent potential active ingredients responsible for inhibiting the proliferation of MDA-MB-231 breast cancer cells.

To better understand their chemical properties and potential biological relevance, these compounds were further categorized based on their structural classes and reported bioactivities, as shown in Table 2. The identified compounds include sesquiterpene lactones (e.g., Arglabin, dehydrocostus lactone, parthenolide), atypical sesquiterpenes (e.g., arteannuin, artemisinic acid), organic acids, and amino acid derivatives. Notably, many of these compounds have been reported to exhibit anti-inflammatory, antitumor, or pro-apoptotic activities, suggesting their potential contribution to the inhibitory effect of EA-2 on MDA-MB-231 breast cancer cells.

### 3.3. Effect of EA-2 on the Viability of Normal Mammary Epithelial Cells

**Figure 3:**
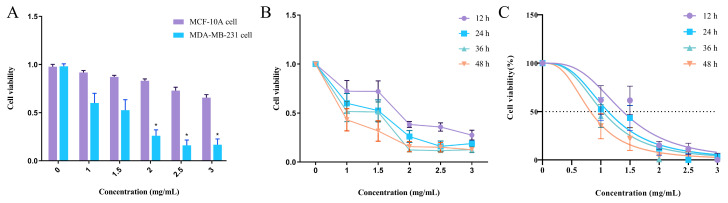
In vitro growth inhibition of MDA-MB-231 breast cancer cells by EA-2. (**A**). Comparison of the growth effects of EA-2 on MDA-MB-231 cells and MCF-10A mammary epithelial cells. (**B**). Concentration–time effect relationship of EA-2 on the growth of MDA-MB-231 cells. (**C**). Half-maximal inhibitory concentration (IC50) of EA-2 on human breast cancer cells at different time points (data are presented as the mean ± SD (*n* = 3), and statistical significance was assessed by two-way ANOVA * *p* < 0.05, vs. control group).

To investigate whether EA-2 could be a potential anti-breast cancer drug and whether it has toxic side effects on normal mammary epithelial cells, the viability of MDA-MB-231 and MCF-10A cells after drug treatment was compared. The cell viability in the MDA-MB-231 group was significantly lower than that in the MCF-10A group (* *p* < 0.05). At a drug concentration of 2 mg/mL, the viability of MDA-MB-231 cells was 0.26 ± 0.06, while the viability of MCF-10A cells was 0.7 ± 0.06, indicating that EA-2 has no toxic side effects on human mammary epithelial cells (Figure 3A).

### 3.4. Concentration–Time Effect of EA-2 on MDA-MB-231 Cell Growth

When MDA-MB-231 breast cancer cells were treated with different drug concentrations, the growth of MDA-MB-231 cells was significantly inhibited as the concentration increased. At a concentration of 1.5 mg/mL, the cell viability of MDA-MB-231 cells at 12 h, 24 h, 36 h, and 48 h was 0.72 ± 0.11, 0.53 ± 0.12, 0.51 ± 0.10, and 0.32 ± 0.11, respectively, indicating that with the extension of treatment time, cell viability gradually decreased (* *p* < 0.05, ** *p* < 0.01). At the same time, the cell viability showed a decreasing trend with increasing drug concentration, as shown in Table 2. When the concentration reached 2.0 mg/mL, further increases in drug concentration (e.g., 2.5 mg/mL) did not result in significant changes in cell viability at the same time points, indicating that 2.0 mg/mL for 24 h was the optimal inhibitory condition (Figure 3B).

### 3.5. EA-2 Inhibits the Proliferation of MDA-MB-231 Breast Cancer Cells In Vitro

EA-2 (0, 1, 1.5, 2, 2.5, 3 mg/mL) was applied to MDA-MB-231 cells for 12, 24, 36, and 48 h, and the cancer cell proliferation ability was assessed by MTT assay. Compared with the control group, EA-2 significantly reduced the survival rate of MDA-MB-231 cells (* *p* < 0.05, ** *p* < 0.01) in a dose-dependent manner. The half-maximal inhibitory concentrations (IC50) were 1.577, 1.124, 1.032, and 0.821 mg/mL, respectively (Figure 3C). As the treatment time increased, the IC50 gradually decreased, but there was no significant difference between the IC50 values at 36 h and 48 h compared to 24 h (*p* > 0.05). This suggests that prolonging the treatment time did not significantly enhance the inhibitory effect of EA-2 (Table 3). For subsequent experiments, a 24 h treatment duration and concentrations of 0.5, 1, 1.5, and 2 mg/mL were selected as the conditions.

### 3.6. Effect of EA-2 on the Morphology of MDA-MB-231 Breast Cancer Cells

The morphology of MDA-MB-231 breast cancer cells was observed under a microscope. The corresponding microscopic images of each treatment group are shown in Supplementary Figure S2. The control group consisted of cells cultured in a normal medium. In contrast, the experimental groups were treated with different concentrations of EA-2 (0.5 mg/mL, 1 mg/mL, 1.5 mg/mL, 2 mg/mL) for 24 h. In the control group, the cells exhibited clear nuclear borders, a rhomboid shape, and tight adherent distribution, growing in clusters with good conditions. Significant morphological changes were observed in the experimental groups with increasing drug concentrations. At 0.5 mg/mL, the cells tended to become more rounded. At 1 mg/mL, intercellular connections weakened, and cell rounding was more pronounced. At 1.5 mg/mL and 2 mg/mL, the cells became distinctly rounded and detached, with intercellular connections almost completely destroyed, resulting in a scattered distribution.

### 3.7. Hoechst Staining Observation of EA-2-Induced Apoptosis in MDA-MB-231 Cells

**Figure 4:**
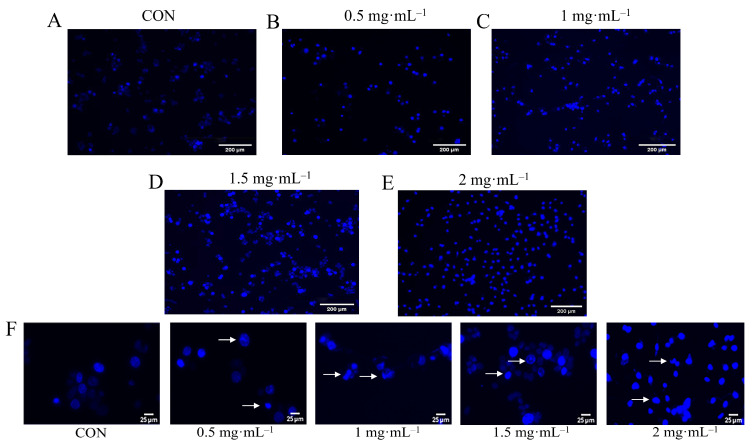
EA-2 increases fluorescence intensity and causes nuclear structural changes in MDA-MB-231 cells. (**A**). Control group. (**B**–**E**). Different concentrations of the EA-2 treatment groups. In the control group, the nuclear morphology is normal with uniform staining. In the treatment groups, after 24 h of treatment, the cells exhibit apoptotic characteristics such as nuclear condensation, chromatin aggregation, and nuclear fragmentation. Scale bar 200 μm. (**F**). Enlarged views of representative nuclear regions at each EA-2 concentration, highlighting typical apoptotic features such as chromatin condensation and fragmentation. Scale bar 25 μm. White arrows indicate nuclear body fragments.

Hoechst staining was used to observe the apoptosis of MDA-MB-231 cells. As shown in Figure 4A–E, the control group exhibited round and oval-shaped nuclei with uniform staining, weak blue fluorescence intensity, and consistent distribution. In contrast, after treatment with 1 mg/mL EA-2, the nuclear morphology showed significant changes, including nuclear condensation, nuclear fragmentation, and chromatin aggregation. Meanwhile, the blue fluorescence intensity was markedly enhanced, indicating that EA-2 treatment could induce apoptosis in MDA-MB-231 cells. To better visualize the nuclear morphological alterations at each concentration, representative enlarged regions are presented in Figure 4F, clearly showing features such as nuclear condensation, chromatin aggregation, and fragmentation. These images provide more intuitive evidence of EA-2 induced apoptosis in a concentration-dependent manner.

### 3.8. Effect of EA-2 on Apoptosis Rate of MDA-MB-231 Cells

**Figure 5:**
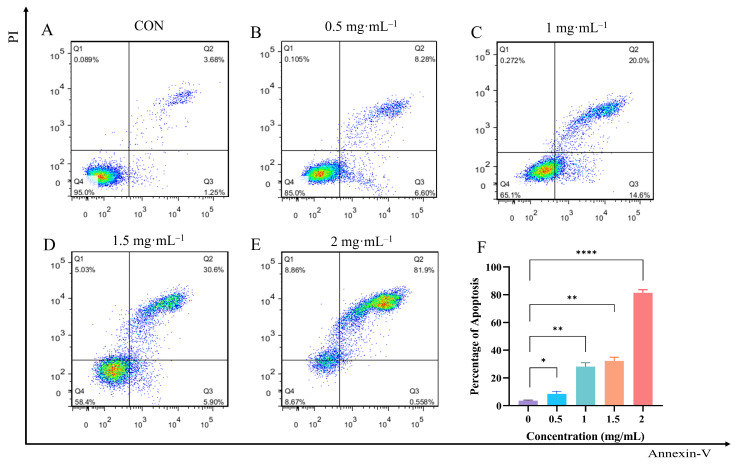
Effect of EA-2 on apoptosis rate of MDA-MB-231 cells at different concentrations. (**A**–**E**). Apoptosis of MDA-MB-231 cells treated with different concentrations of EA-2. (**F**). Statistical analysis of apoptosis results. (Data are presented as the mean  ±  SD (*n*  =  3), and statistical significance was assessed by one-way ANOVA. * *p* < 0.05, ** *p* < 0.01, **** *p* < 0.0001, vs. control group).

Cell apoptosis was detected using the Annexin V-FITC/PI dual staining method. After 24 h of treatment with different concentrations, apoptosis in MDA-MB-231 cells was analyzed by flow cytometry. The results, as shown in Figure 5A–E, indicate that EA-2 significantly affects the apoptosis of MDA-MB-231 cells. As the concentration increased, the proportions of early (Q2 region) and late apoptotic cells (Q3 region) gradually increased, while the proportion of live cells (Q4 region) decreased. This suggests that EA-2 effectively induces apoptosis in MDA-MB-231 cells at higher concentrations. The results (Figure 5F) further show that the concentration of EA-2 is positively correlated with the percentage of apoptosis, and this indicates that the anti-apoptotic effect of EA-2 is concentration-dependent. Statistical analysis shows significant differences between different concentration groups (* *p* < 0.05), confirming that EA-2 induces apoptosis in breast cancer cells.

### 3.9. Effect of EA-2 on Cell Cycle Distribution of MDA-MB-231 Cells

**Figure 6:**
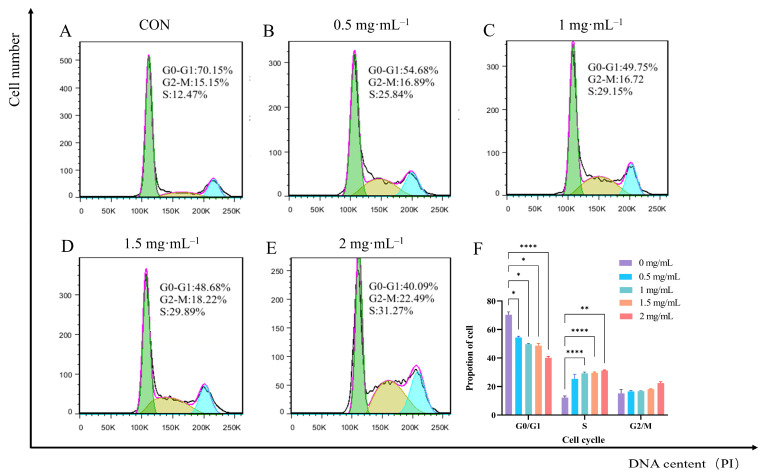
Impact of different concentrations of EA-2 on the cell cycle distribution of MDA-MB-231 cells. (**A**–**E**). Distribution of MDA-MB-231 cells in the cell cycle under different concentrations of EA-2 treatment. (**F**). Statistical analysis of cell cycle distribution. (Data are presented as the mean  ±  SD (*n*  =  3), and statistical significance was assessed by two-way ANOVA * *p* < 0.05, ** *p* < 0.01, **** *p* < 0.0001, vs. control group).

Flow cytometry results (Figure 6A–E) show that after treatment with different concentrations of EA-2, the cell cycle distribution of MDA-MB-231 cells was significantly altered. Compared with the control group, all dose groups reduced the proportion of cells in the G0/G1 phase (* *p* < 0.05) and increased the proportion of cells in the S phase (* *p* < 0.05) and G2/M phase. However, the increase in the G2/M phase cell population was not statistically significant (*p* > 0.05), suggesting that EA-2 induces cell cycle arrest in the S and G2/M phases (Figure 6F). EA-2 inhibits cell proliferation to some extent by altering the cell cycle distribution of MDA-MB-231 cells, particularly by blocking the cells in the S phase, indicating its potential anticancer effect.

### 3.10. Differential Protein Identification and Quantification Analysis

**Figure 7:**
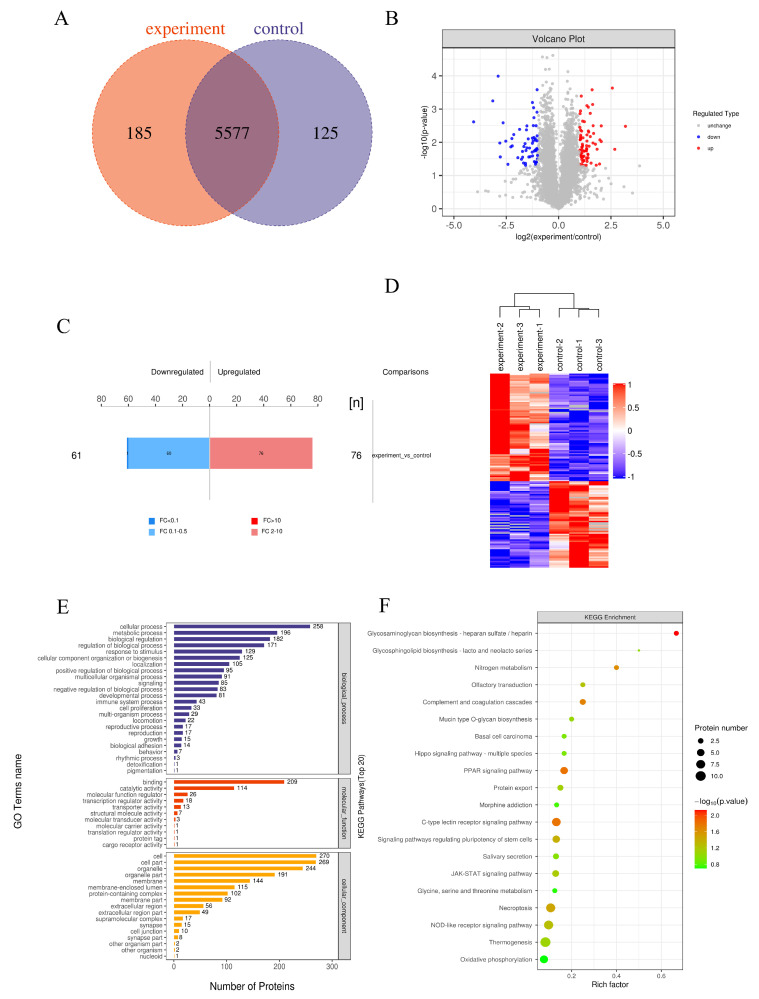
Differential protein expression analysis. (**A**). Statistical chart of protein expression differences between groups. (**B**). Volcano plot of protein expression differences between the experimental and control groups. (**C**). Bar chart of quantitative protein expression differences. (**D**). Clustering analysis of differentially expressed proteins. (**E**). GO annotation analysis of differentially expressed proteins. (**F**). KEGG metabolic pathway enrichment analysis of differentially expressed proteins.

We performed label-free quantitative proteomics to analyze the differential protein expression between EA-2-treated MDA-MB-231 cells and untreated MDA-MB-231 breast cancer cells. Mass spectrometry data were analyzed using MaxQuant software (v1.5.3.17), with both protein and peptide identifications controlled at a false discovery rate (FDR) of ≤ 0.01. A total of 5577 proteins were identified. Differentially expressed proteins (DEPs) were defined by a fold change > 2 or < 0.5 (i.e., |log_2_FC| ≥ 1) and a *p*-value < 0.05. Based on these criteria, 137 DEPs were identified, including 76 upregulated and 61 downregulated proteins (Figure 7A–C). Hierarchical clustering analysis revealed significant differences between the groups (Figure 7D). Gene Ontology (GO) analysis (Figure 7E) showed that these differential proteins were primarily localized to the cell membrane and organelles, as well as involved in metabolic and regulatory pathways. Kyoto Encyclopedia of Genes and Genomes (KEGG) pathway enrichment analysis (Figure 7F) indicated that the differential proteins were mainly enriched in the PPAR signaling pathway, JAK-STAT signaling pathway, PI3K-Akt signaling pathway, and MAPK signaling pathway. The cooperative effect of multiple signaling pathways stimulates apoptosis and inhibits proliferation in breast cancer cells. Subsequently, the expression levels of differentially expressed proteins—including PI3K, AKT1S1, SIRT6, JAK1, SCD, STAT3, CASP8, STAT6, PAK1, and FABP4—were significantly downregulated. These results suggest that EA-2 may induce apoptosis and inhibit cell proliferation in breast cancer cells through the coordinated action of multiple signaling pathways.

### 3.11. Validation Analysis of Interactions Between Major Active Compounds and Potential Targets

Based on KEGG pathway enrichment analysis, the JAK-STAT, PI3K-Akt, and PPAR signaling pathways were identified as key regulatory pathways. To further validate the interactions between active compounds and core targets, molecular docking was performed between the major bioactive constituents of dandelion and key differential proteins within these pathways, including PI3K, PAK1, JAK1, STAT3, and FABP4. The results demonstrated that the binding energies of most compound–target interactions were below −5 kcal·mol^−1^ (Table 4), suggesting strong binding affinities. Among the compounds, Arteannuin, Atractylenolide II, and Arglabin exhibited particularly strong binding capabilities. Structural analysis revealed that these three compounds have relatively low molecular weights—282.3, 327.4, and 246.3 Da, respectively—and all belong to the class of terpenoids. This structural feature may contribute to their enhanced ability to bind target active sites and penetrate cellular membranes.

Furthermore, analysis of their lipid–water partition coefficients (LogP) showed values of 2.93, 2.37, and 4.12, respectively, all falling within the optimal range of 2–5. This suggests favorable lipophilicity for tissue permeability and potential bioavailability in tumor environments. Notably, previous studies have confirmed the significant antitumor effects of Arteannuin and Arglabin, with derivatives of Arglabin already approved for clinical treatment of breast cancer in Kazakhstan. The results suggest that these compounds may represent the principal active constituents in dandelion responsible for its inhibitory effects on breast cancer. This finding is highly consistent with the KEGG pathway enrichment results, further indicating that these compounds may exert their biological effects by modulating the identified signaling pathways.

### 3.12. MTT Evaluation of Bioactive Compounds on MDA-MB-231 Breast Cancer Cells

**Figure 8:**
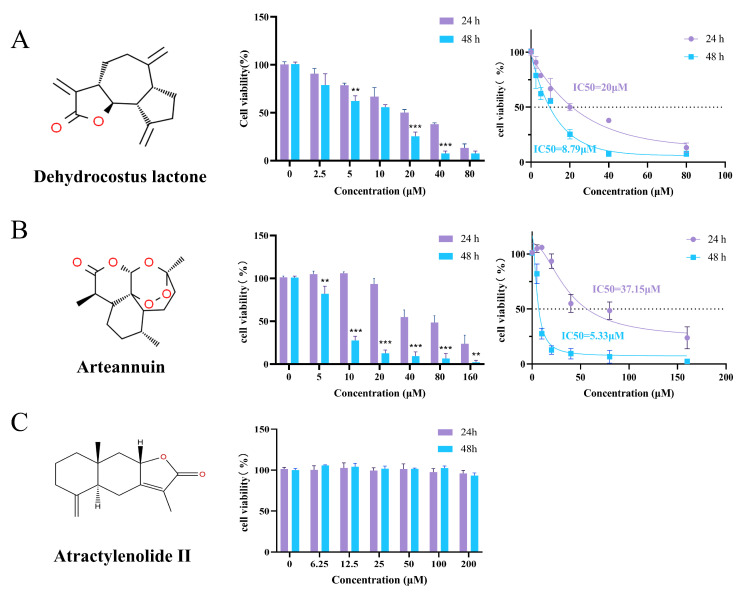
Inhibitory effects of three representative compounds identified from EA-2 on the viability of MDA-MB-231 cells. (**A**). Dehydrocostus lactone. (**B**). Arteannuin. (**C**). Atractylenolide II. They were evaluated for their antiproliferative activity using MTT assays at 24 h and 48 h. (Data are presented as the mean  ± SD (*n* = 3), and statistical significance was assessed by two-way ANOVA, ** *p* < 0.01, *** *p* < 0.001, vs. control group).

To further validate the bioactivity of the identified compounds, Dehydrocostus lactone, Arteannuin, and Atractylenolide II were selected for individual testing based on their superior molecular docking results. MTT assays were performed to determine their inhibitory effects on the proliferation of MDA-MB-231 cells. The results showed that Dehydrocostus lactone exhibited strong cytotoxicity against MDA-MB-231 cells in a dose- and time-dependent manner, with IC_50_ values of 20 μM at 24 h and 8.79 μM at 48 h (Figure 8A). Arteannuin also demonstrated significant inhibition in a dose- and time-dependent manner, with IC_50_ values of 37.15 μM (24 h) and 5.33 μM (48 h) (Figure 8B). In contrast, Atractylenolide II showed no obvious inhibitory effect on cell viability under the same conditions, indicating a weaker impact on breast cancer cell viability and suggesting it is not a major antitumor active compound (Figure 8C).

In addition to the three tested compounds, the remaining nine compounds identified in the EA-2 composition—Arglabin, Parthenolide, Isoalantolactone, Linderalactone, Artemisinic acid, (R)-Mandelic acid, Azelaic acid, Nicotinic acid, and Betaine—also possess reported antitumor activities, particularly against breast cancer and MDA-MB-231 cells. For example, Arglabin has been found to exert antitumor effects by inhibiting farnesyltransferase and has been approved in Kazakhstan as an anticancer drug for the treatment of breast cancer, ovarian cancer, and lung cancer [32]. Parthenolide may inhibit angiogenesis by reducing the secretion of angiogenic factors from MDA-MB-231 breast cancer cells, thereby interfering with endothelial cell proliferation, migration, and tube formation, ultimately suppressing tumor growth [33]. Isoalantolactone and Linderalactone, both sesquiterpene lactones, exhibit inhibitory effects on triple-negative breast cancer [34,35]. Artemisinic acid, as an artemisinin-type sesquiterpene, inhibits breast cancer cell growth in a dose-dependent manner [36]. Although small molecule organic acids such as (R)-Mandelic acid, Azelaic acid, and Nicotinic acid are not classical anticancer agents, their reported anti-inflammatory, antimicrobial, or antioxidant properties may help modulate the tumor microenvironment or enhance the bioavailability of other active compounds.

In summary, Dehydrocostus lactone, Arteannuin, and Arglabin are likely the primary active components responsible for the antiproliferative effects of EA-2. While the other compounds vary in potency and mechanisms, they may play supportive roles in the overall effect. This interplay reflects the intrinsic complexity of EA-2 and supports its potential as a multi-component therapeutic candidate for breast cancer intervention.

## 4. Discussion

Breast cancer is the most commonly diagnosed malignant tumor among women worldwide and poses a serious threat to women’s health. Epidemiological data indicate a continuous increase in its incidence, making breast cancer the leading cause of cancer-related mortality among women [37]. Among them, specific molecular subtypes such as TNBC lack effective targeted therapeutic strategies, and the development of acquired resistance induced by chemotherapy and targeted therapies further reduces treatment response rates [38]. It is noteworthy that conventional chemotherapeutic agents are often associated with dose-limiting toxicities, including bone marrow suppression and cardiotoxicity, which severely affect patients’ treatment tolerance and long-term prognosis [39]. Therefore, there is an urgent need to identify safer and more effective adjuvant therapeutic agents, which has become a pressing priority in the field of breast cancer treatment.

Existing studies have confirmed that dandelion exhibits multiple biological activities, including anti-inflammatory, antioxidant, and immunomodulatory effects. Its polyphenols and flavonoids exert significant antitumor effects in various malignancy models by regulating cell cycle arrest, inducing apoptosis, and inhibiting epithelial–mesenchymal transition (EMT) [40,41]. However, the precise molecular mechanisms and regulatory networks of dandelion extracts against breast cancer cells remain incompletely understood, and its application in precision breast cancer therapy requires systematic preclinical validation. In view of this, the present study further systematically investigated the selective inhibitory effects and molecular mechanisms of dandelion extracts on TNBC MDA-MB-231 cells. The results demonstrated that dandelion root alcohol extracts significantly inhibited the proliferation of MDA-MB-231 cells, with the ethyl acetate (EA-2) fraction exhibiting the strongest antitumor activity, suggesting that this fraction may be enriched with the principal active components against breast cancer. LC-MS analysis successfully identified 12 potential active compounds, mainly including sesquiterpene lactones (e.g., Arglabin, dehydrocostus lactone, parthenolide), atypical sesquiterpenes (e.g., arteannuin, artemisinic acid), organic acids, and amino acid derivatives.

Among them, Isoalantolactone significantly inhibits human breast cancer cell proliferation, and its activity is related to the α-methylene-γ-lactone group in its molecular structure, which can interfere with DNA replication in cancer cells through alkylation [42,43]. Artemisinin exerts inhibitory effects on TNBC MDA-MB-231 cells by generating reactive oxygen species (ROS) via iron-mediated oxidative stress, effectively inducing apoptosis [44]. Parthenolide can inhibit the NF-κB pathway, reducing inflammatory factor release and indirectly suppressing the tumor microenvironment [45,46]. Importantly, the extract effectively inhibits tumor cells without obvious toxicity to MCF-10A normal mammary epithelial cells, demonstrating good selective inhibition. The MTT assay accurately assessed the inhibitory effect on cell proliferation, showing that within a low concentration range (0.5–2 mg/mL), dandelion extract did not significantly affect normal cell viability (*p* > 0.05), whereas it exhibited significant inhibitory effects on MDA-MB-231 breast cancer cells at equivalent concentrations, suggesting that dandelion extract may reduce the side effects of conventional chemotherapy on normal tissues.

Cell cycle analysis using PI staining combined with flow cytometry revealed significant changes in cell cycle distribution in MDA-MB-231 cells after 24 h treatment with EA-2, with notable increases in the proportions of cells in S and G2/M phases. This indicates that EA-2 may induce DNA damage accumulation and S phase arrest by inhibiting key enzymes involved in DNA synthesis such as DNA polymerase or topoisomerase, or by inducing replication stress. Previous studies have confirmed that anticancer drugs can block mitotic progression by inhibiting the activity of the CDK1/Cyclin B complex, thereby inducing G2/M phase arrest, leading to a significant increase in the G2/M population and suppressing breast cancer cell proliferation [47,48,49,50]. Additionally, G2/M arrest may enhance cellular sensitivity to DNA damage signals, further promoting apoptosis in cancer cells [51].

LFQ is a widely used high-throughput protein analysis technique in biomedical mechanism research [52]. This study applied label-free quantitative proteomics to systematically analyze the effects of EA-2 treatment on the protein expression profile of triple-negative breast cancer MDA-MB-231 cells. Mass spectrometry detected a total of 5577 proteins, among which 137 showed significant differential expression. Among them, 76 proteins were upregulated, including RIT1 and FABP4, while 61 proteins were downregulated, involving key regulatory molecules such as PI3K, AKT1S1, SIRT6, JAK1, SCD, STAT3, and CASP8. Bioinformatics analysis revealed that these differentially expressed proteins were significantly enriched in three key KEGG pathways. In the PI3K-Akt signaling pathway, upstream regulators RIT1 and PAK1 promote activation of PI3K/Akt, thereby regulating cell survival and cell cycle progression [53,54]. We observed significant upregulation of upstream regulatory factors such as PAK1 and RIT1, while mTORC1 negative regulator AKT1S1 and metabolic regulator SIRT6 were downregulated, suggesting that EA-2 may inhibit cell proliferation and induce programmed cell death by interfering with the pro-survival PI3K-Akt-mTOR signaling axis. In the JAK-STAT signaling pathway, JAK1 indirectly regulates cell proliferation and apoptosis through activation of downstream STAT factors; the significant downregulation of STAT family member STAT3 and apoptosis-related protein CASP8 indicates that EA-2 may suppress tumor cell proliferation and anti-apoptotic capacity by blocking JAK-STAT3 signaling transduction. In the PPAR signaling pathway, the upregulation of PPARγ and its target gene FABP4, along with the significant downregulation of lipid synthesis enzyme SCD, reveals that EA-2 may remodel tumor cell lipid metabolism by activating the PPARγ pathway [55,56]. This study systematically reveals that the active components in dandelion extract EA-2 may achieve multi-dimensional regulation of proliferation, apoptosis, and metabolic reprogramming in MDA-MB-231 breast cancer cells through multi-target and multi-pathway mechanisms. These findings not only provide a solid scientific basis for the application of dandelion in breast cancer treatment but also lay an important theoretical foundation for the development of multi-pathway synergistic therapies targeting triple-negative breast cancer.

## 5. Conclusions

This study focused on the inhibitory effects of dandelion extract on breast cancer cells and systematically explored its impact on the proliferation, apoptosis, and cell cycle of MDA-MB-231 cells, along with its underlying molecular mechanisms. The results demonstrated that the dandelion root extract exhibited significant anti-proliferative effects against breast cancer cells, with the EA-2 fraction effectively inhibiting MDA-MB-231 cell proliferation, inducing cell cycle arrest and promoting apoptosis. Proteomics-based analysis revealed that EA-2 exerts its effects by modulating the JAK-STAT, PI3K-Akt, and PPAR signaling pathways, as well as lipid metabolism-related pathways, thereby synergistically suppressing proliferation and promoting apoptosis in breast cancer cells. The underlying mechanisms involve the regulation of key proteins such as PI3K, PAK1, JAK1, SIRT6, and FABP4, disrupting the survival capacity and metabolic homeostasis of MDA-MB-231 cells. This study provides a theoretical foundation for understanding the molecular mechanisms underlying the anti-breast cancer effects of dandelion extract and offers an experimental basis and reference for the development of novel therapeutic strategies for breast cancer treatment.

## Figures and Tables

**Table 1:** Mass spectrometry identification results of potential compounds in EA-2.

No.	Compound	Rt (min)	Molecular Formula	Quantifier Ion		mzVault Best Match
				Type	*m*/*z*	
1	Betaine	0.587	C_5_H_11_NO_2_	[M + H]^+^	118.0865	84.6
2	(R)-Mandelic acid	3.027	C_8_H_8_O_3_	[M − H]^−^	151.0399	77.9
3	Azelaic acid	5.057	C_9_H_16_O_4_	[M − H]^−^	187.0974	85.3
4	Arglabin	5.806	C_15_H_18_O_3_	[M + H]^+^	247.1329	76.7
5	Dehydrocostus lactone	5.258	C_15_H_18_O_2_	[M + H]^+^	231.1380	93.5
6	Arteannuin	6.071	C_15_H_20_O_3_	[M + H]^+^	249.1479	83.5
7	Nicotinic acid	0.819	C_6_H_5_NO_2_	[M + H]^+^	124.0395	80.6
8	Atractylenolide II	4.757	C_15_H_20_O_2_	[M + H]^+^	233.1532	83.7
9	Parthenolide	6.068	C_15_H_20_O_3_	[M + H]^+^	249.1099	90.3
10	Linderalactone	4.699	C_15_H_16_O_3_	[M + H]^+^	245.1172	89.0
11	Artemisinic acid	5.261	C_15_H_22_O_2_	[M + H]^+^	235.1693	84.6
12	Isoalantolactone	4.762	C_15_H_20_O_2_	[M − H]^−^	233.1535	86.2

**Table 2:** Major compound categories and their bioactivities.

Category	Members	Main Biological Activities
Sesquiterpene lactones	Arglabin, Dehydrocostus lactone, Atractylenolide II, Parthenolide, Isoalantolactone, Linderalactone	Anti-inflammatory [15,16], antitumor [17,18,19], apoptosis induction [20,21]
Sesquiterpenes (atypical)	Arteannuin, Artemisinic acid	Antimalarial [22], anti-inflammatory [23,24], anticancer [25]
Organic acids	(R)-Mandelic acid, Azelaic acid, Nicotinic acid	Antibacterial [26], anti-inflammatory [27,28]
Amino acid derivatives	Betaine	Cytoprotective [29], antioxidant [30], metabolic regulation [31]

**Table 3:** Comparison of cell viability and IC50 values over time ($\bar{x}$ ± S, *n* = 3).

Time (hours)	Cell Viability	IC50 (mg/mL)
12	0.72 ± 0.11	1.577 ± 0.23
24	0.53 ± 0.12 *	1.124 ± 0.18 *
36	0.51 ± 0.10 *	1.032 ± 0.12 *
48	0.32 ± 0.11 **	0.821 ± 0.20 *

Data are presented as the mean ± SD (*n* = 3), and statistical significance was assessed by one-way ANOVA. * *p* < 0.05, ** *p* < 0.01, vs. 12 h.

**Table 4:** Molecular docking results of major active compounds in EA-2 with potential targets.

Active Compounds	Binding Energy/ (kcal·mol^−1^)				
	PI3K	PAK1	JAK1	STAT3	FABP4
Betaine	−4.2	−3.2	−3.6	−3.5	−4.5
(R)-Mandelic acid	−6.6	−5.3	−5.9	−5.3	−4.6
Azelaic acid	−5.9	−4.8	−3.5	−5.4	−4.2
Arglabin	−7.9	−6.1	−8.7	−6.7	−8.6
Dehydrocostus lactone	−7.8	−6.9	−8.5	−6.7	−7.0
Arteannuin	−8.4	−7.3	−9.3	−7.5	−8.9
Nicotinic acid	−5.6	−3.8	−4.8	−4.6	−4.9
Atractylenolide II	−8.2	−6.8	−7.8	−6.8	−6.5
Parthenolide	−7.9	−4.2	−7.5	−6.7	−7.4
Linderalactone	−8.1	−6.6	−7.8	−6.6	−8.1
Artemisinic acid	−7.5	−6.1	−7.2	−6.6	−5.5
Isoalantolactone	−8.3	−6.8	−8.6	−6.4	−6.1

## Data Availability

The data supporting this study’s findings are available from the corresponding authors upon reasonable request.

## Supplementary Materials

The following supporting information can be downloaded at https://www.mdpi.com/article/10.3390/biology14080910/s1, Supplementary Figure S1: Untargeted qualitative analysis and identification of herbal chemical constituents based on LC-MS. Supplementary Figure S2: EA-2-induced growth inhibition and morphological changes in MDA-MB-231 breast cancer cells. Supplementary Table S1: MaxQuant Identification and Quantification Parameters.

## Abbreviations

The following abbreviations are used in this manuscript:

LC-MS	Liquid chromatography–tandem mass spectrometry
TNBC	Triple-negative breast cancer
ER	Estrogen receptor
PR	Progesterone receptor
HER2	Human epidermal growth factor receptor 2
CDKs	Cyclin-dependent kinases
PCD	Programmed cell death
FBS	Fetal bovine serum
ESI	Electrospray ionization
TIC	Total ion chromatograms
HRMS	Human epidermal growth factor receptor 2
IC50	Half-maximal inhibitory concentration
PI	Propidium iodide
LFQ	Label-Free Quantification
DR-AE	Dandelion root aqueous extract
DR-EE	Dandelion root ethanol extract
DL-AE	Dandelion leaf aqueous extract
DL-EE	Dandelion leaf ethanol extract
EA	Ethyl acetate
EA-2	The second subfraction of the ethyl acetate extract
FDR	False discovery rate
DEPs	Differentially expressed proteins
GO	Gene Ontology
KEGG	Kyoto Encyclopedia of Genes and Genomes
LogP	Partition coefficients
EMT	Epithelial–mesenchymal transition
ROS	Reactive oxygen species
UPLC	Ultra-performance liquid chromatography
SIM	Selected ion monitoring
SDT	Lysis buffer composed of SDS, DTT, and Tris-HCl
SDS	Sodium dodecyl sulfate
DTT	Dithiothreitol
BCA	Bicinchoninic Acid Assay
FASP	Filter-aided sample preparation
IAA	Iodoacetamide
DDA	Data-dependent acquisition
HCD	Higher-energy collisional dissociation
FC	Fold change
KAAS	KEGG Automatic Annotation Server
