# Supplementary material for: Mechanistic Study on the Inhibitory Effect of Dandelion Extract on Breast Cancer Cell Proliferation and Its Induction of Apoptosis
Source: Biology (Basel). 2025 Jul 22;14(8):910. doi: 10.3390/biology14080910 (PMC12383382; doi:10.3390/biology14080910)
Supplement: Supplementary file 1 [file biology-14-00910-s001.zip › biology-3700537-supplementary.pdf]

A

Betaine

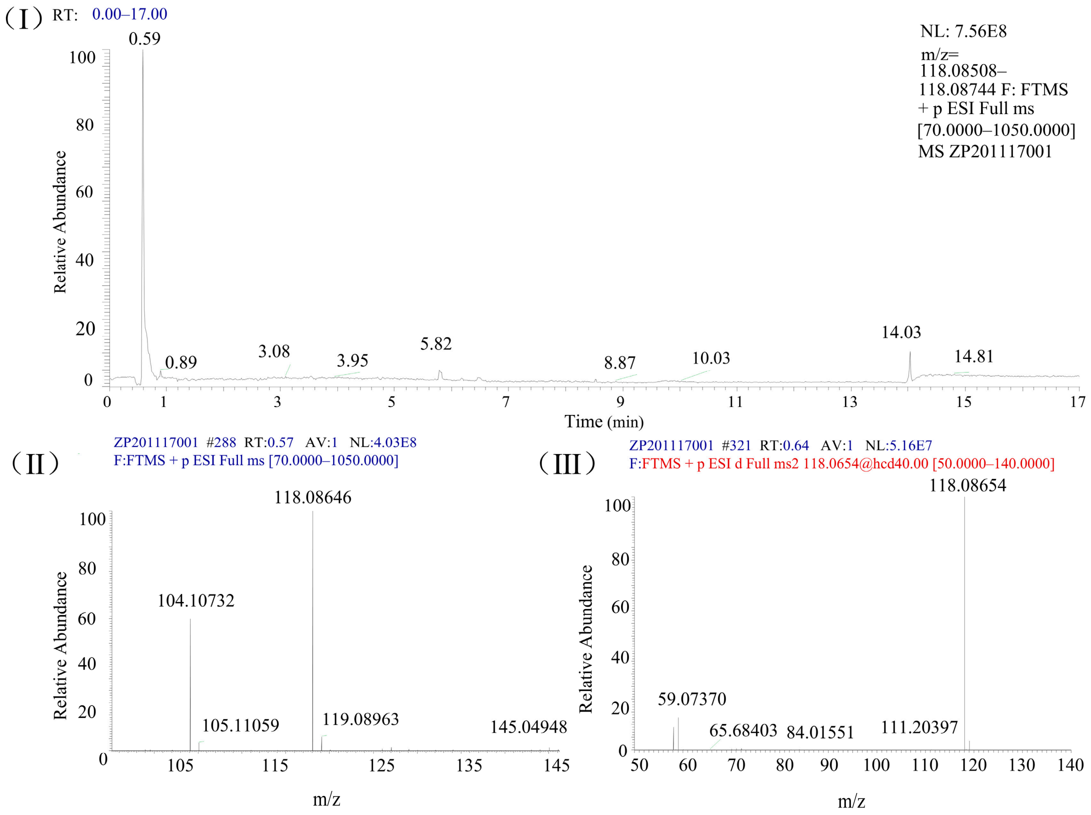

B

(R)-Mandelic acid

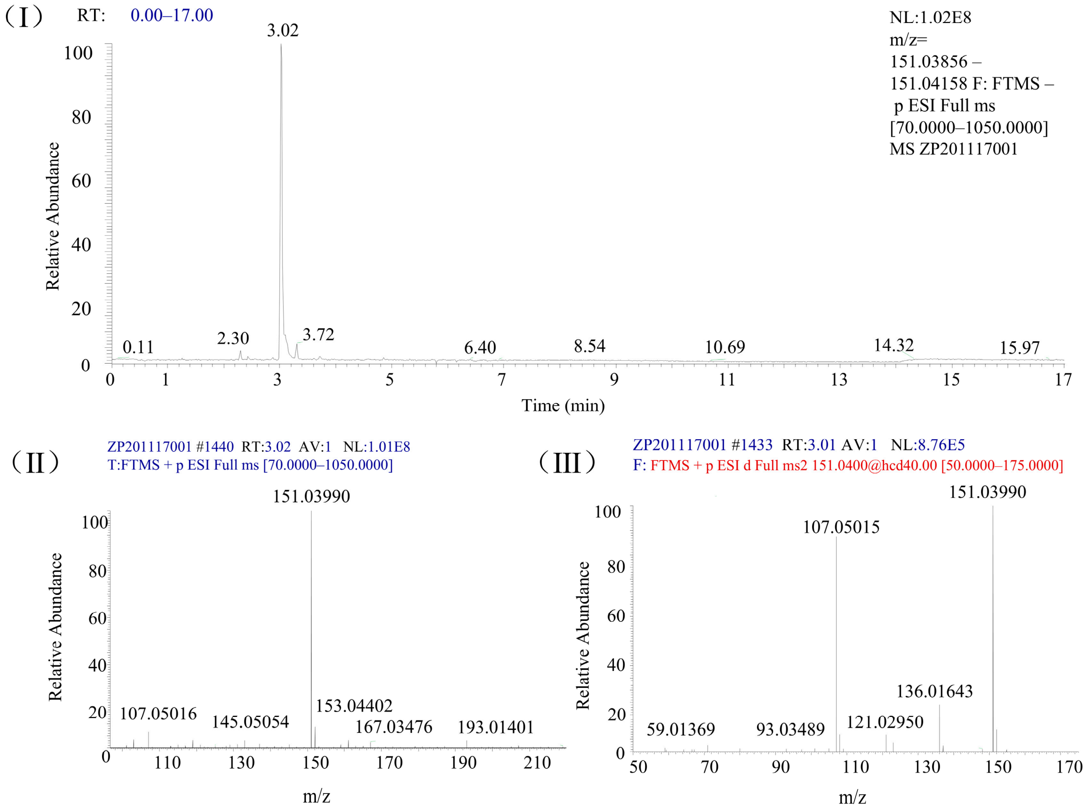

C

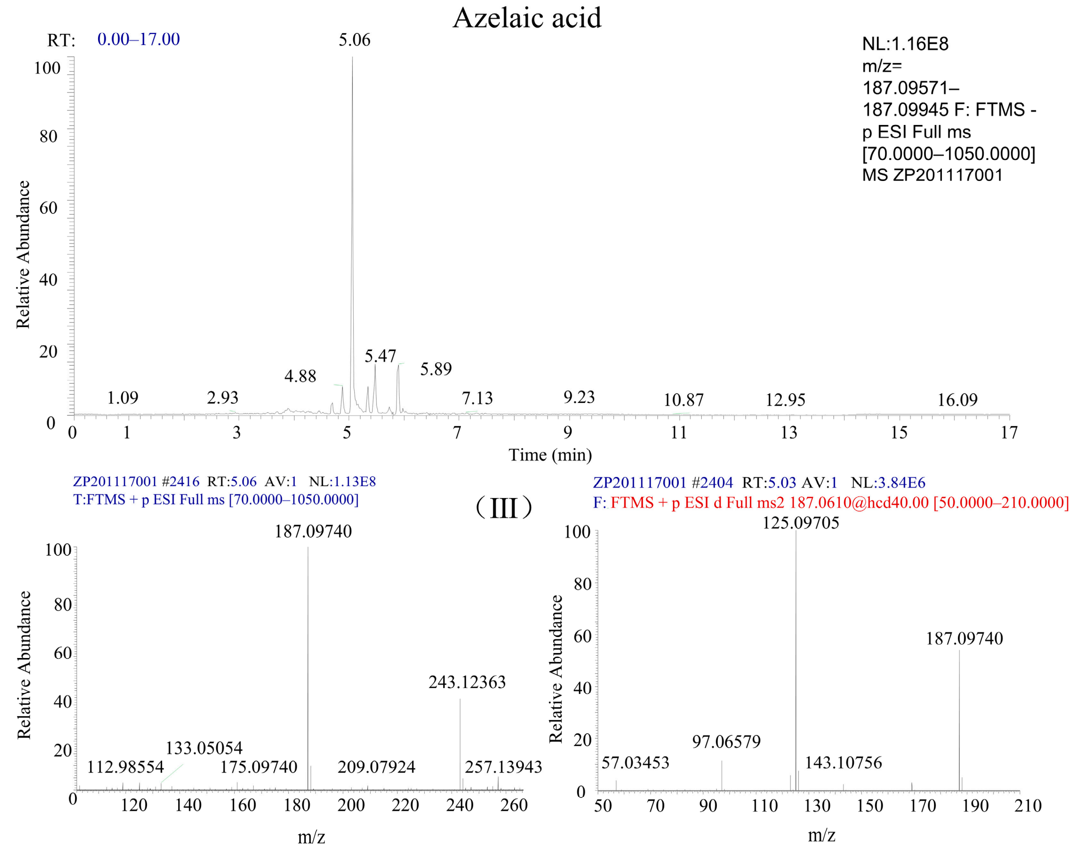
Azelaic acid
(I)
RT: 0.00–17.00
NL:1.16E8
m/z=
187.09571–
187.09945 F: FTMS -
p ESI Full ms
[70.0000–1050.0000]
MS ZP201117001
5.06
5.47
5.89
4.88
1.09
2.93
7.13
9.23
10.87
12.95
16.09
Relative Abundance
Time (min)
(II)
ZP201117001 #2416 RT:5.06 AV:1 NL:1.13E8
T:FTMS + p ESI Full ms [70.0000–1050.0000]
187.09740
243.12363
133.05054
112.98554
175.09740
209.07924
257.13943
Relative Abundance
m/z
(III)
ZP201117001 #2404 RT:5.03 AV:1 NL:3.84E6
F: FTMS + p ESI d Full ms2 187.0610@hcd40.00 [50.0000–210.0000]
125.09705
187.09740
97.06579
57.03453
143.10756
Relative Abundance
m/z

D

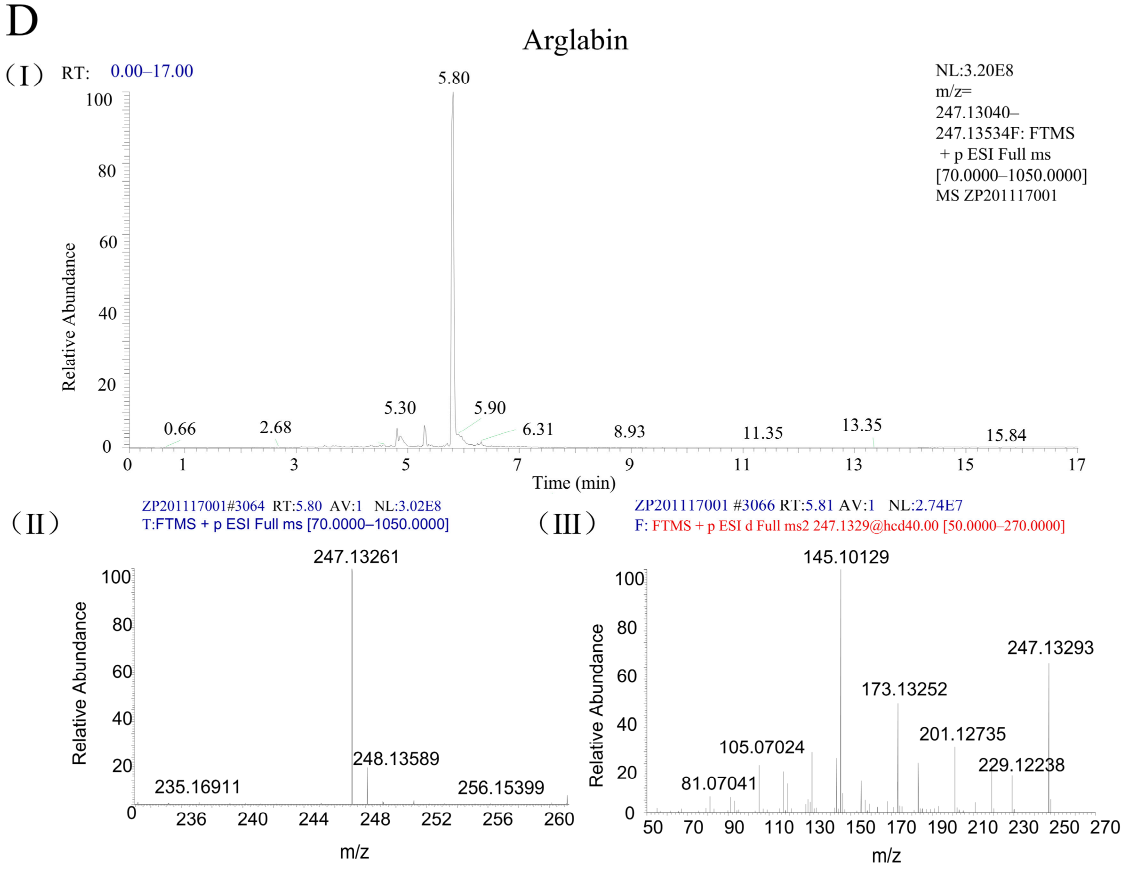
Arglabin
(I)
RT: 0.00–17.00
NL:3.20E8
m/z=
247.13040–
247.13534F: FTMS
+ p ESI Full ms
[70.0000–1050.0000]
MS ZP201117001
5.80
5.30
5.90
6.31
0.66
2.68
8.93
11.35
13.35
15.84
Relative Abundance
Time (min)
(II)
ZP201117001#3064 RT:5.80 AV:1 NL:3.02E8
T:FTMS + p ESI Full ms [70.0000–1050.0000]
247.13261
248.13589
235.16911
256.15399
Relative Abundance
m/z
(III)
ZP201117001 #3066 RT:5.81 AV:1 NL:2.74E7
F: FTMS + p ESI d Full ms2 247.1329@hcd40.00 [50.0000–270.0000]
145.10129
247.13293
173.13252
201.12735
105.07024
229.12238
81.07041
Relative Abundance
m/z

E

Dehydrocostus lactone

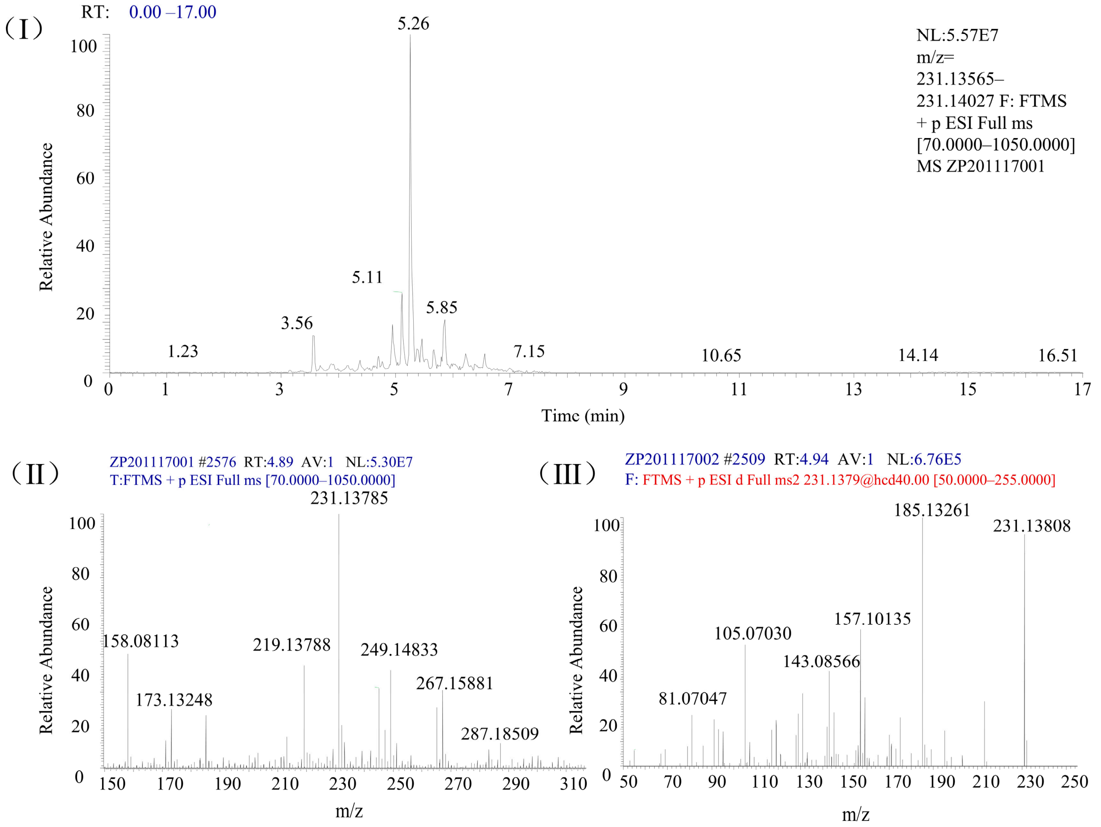

F

Arteannuin

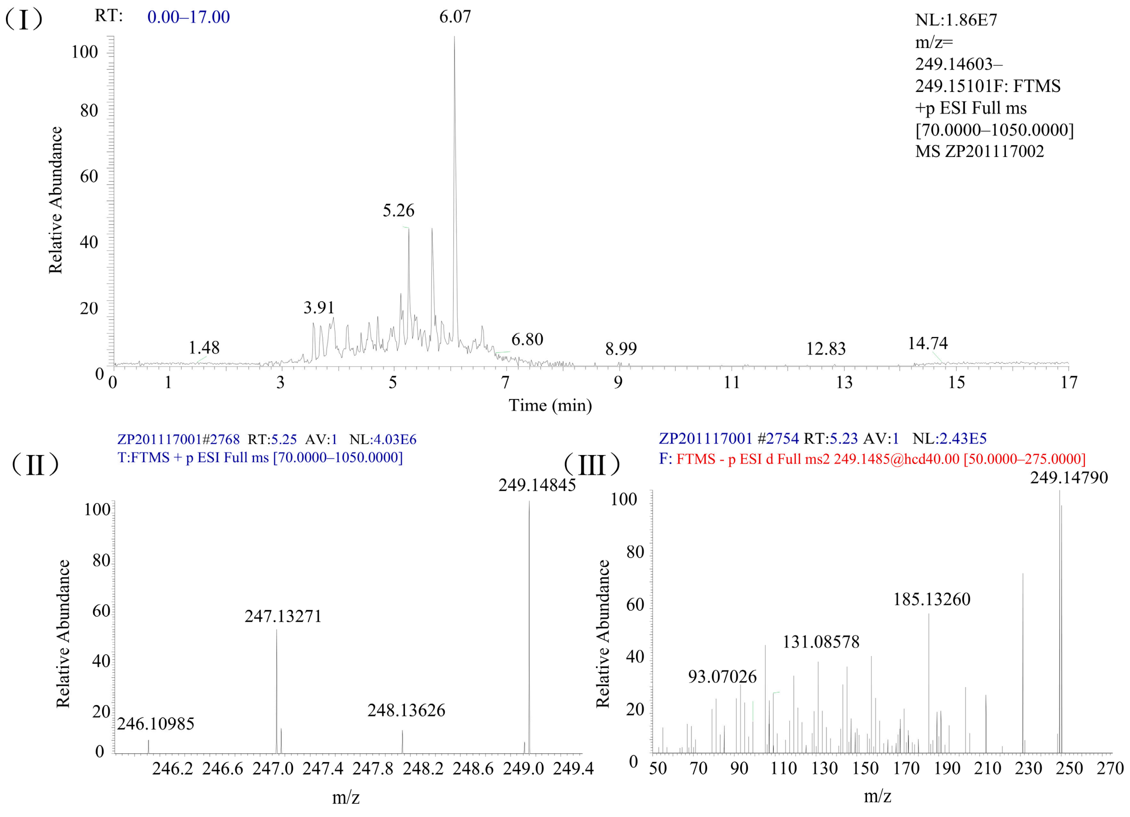

G

Nicotinic acid

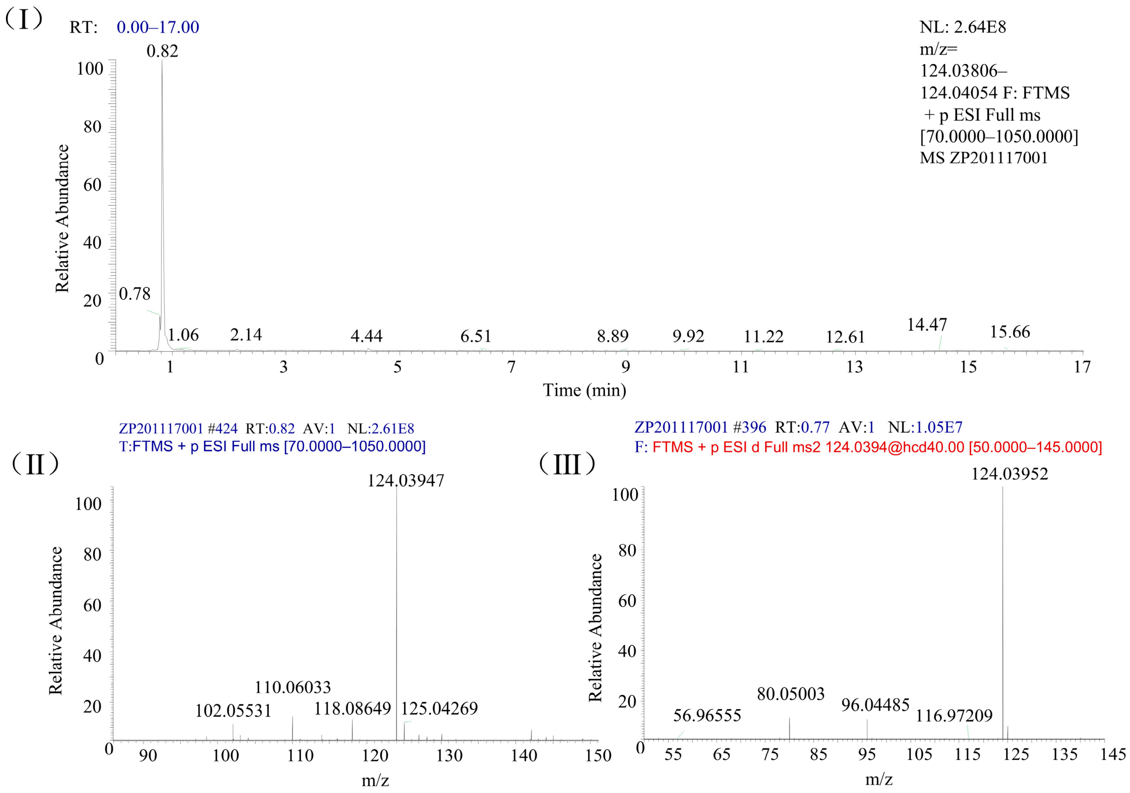

H

Atractylenolide II

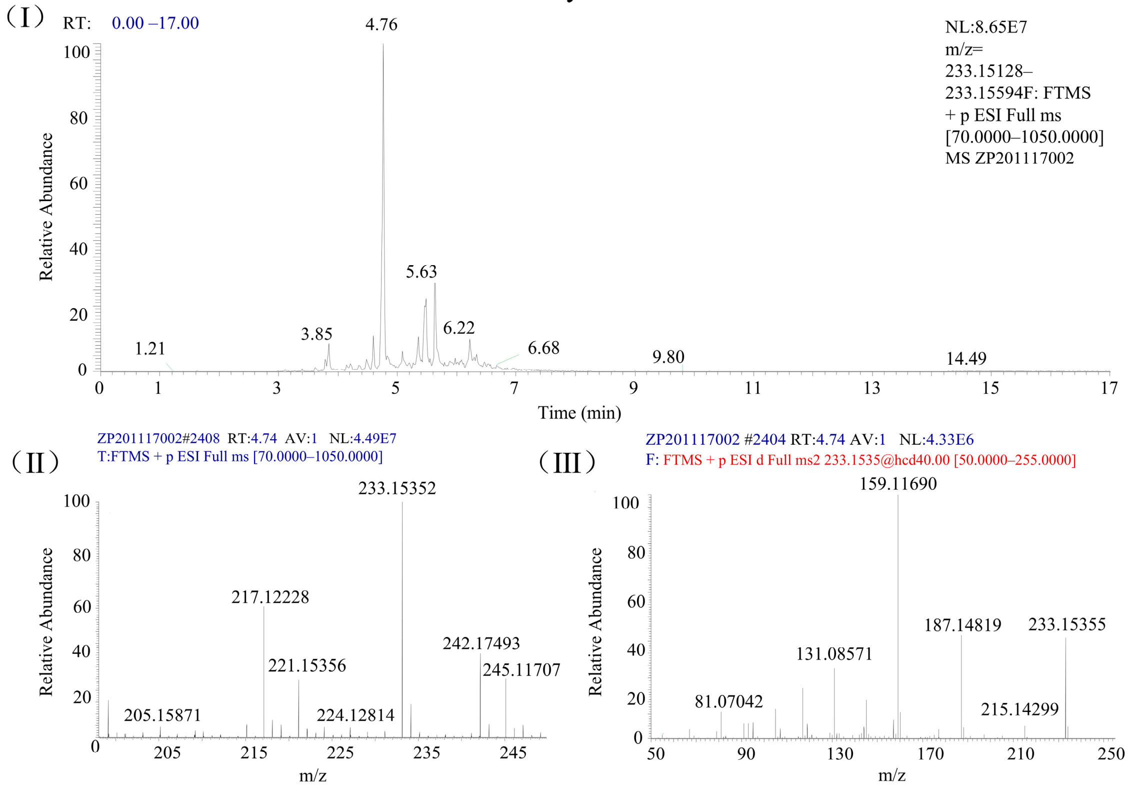

I

Parthenolide

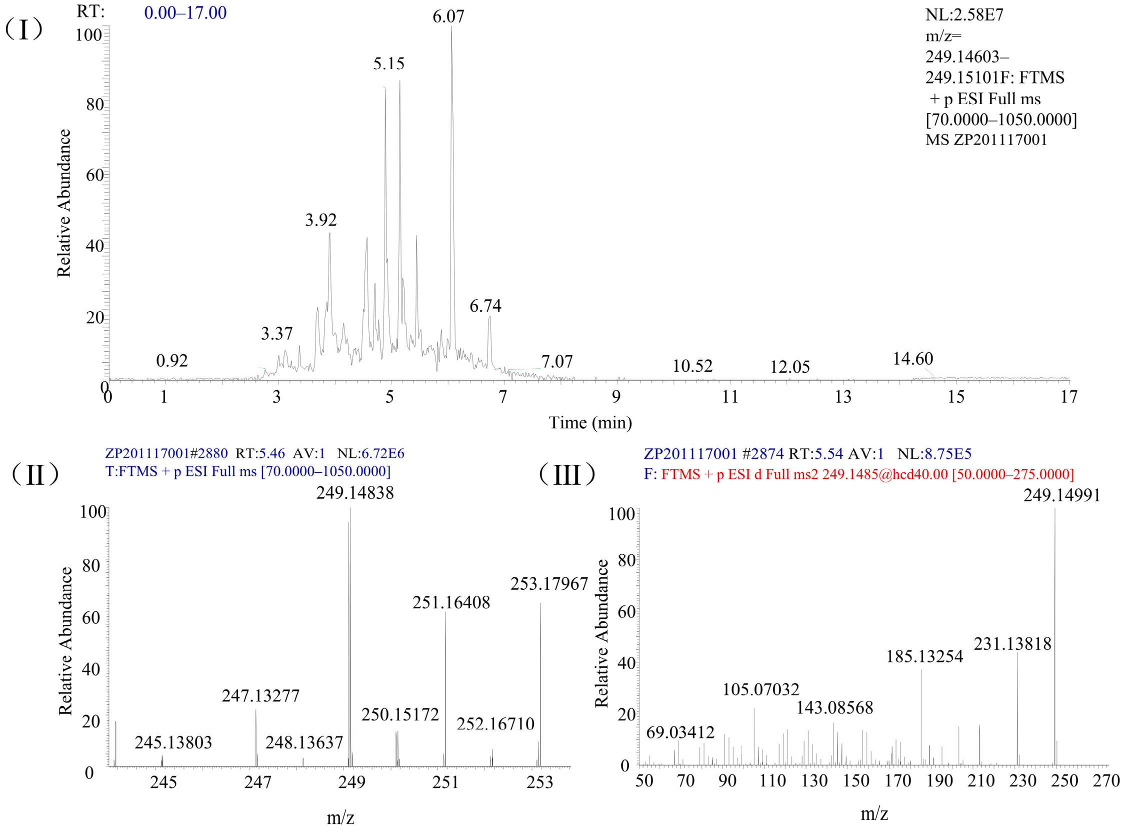

J

Linderalactone

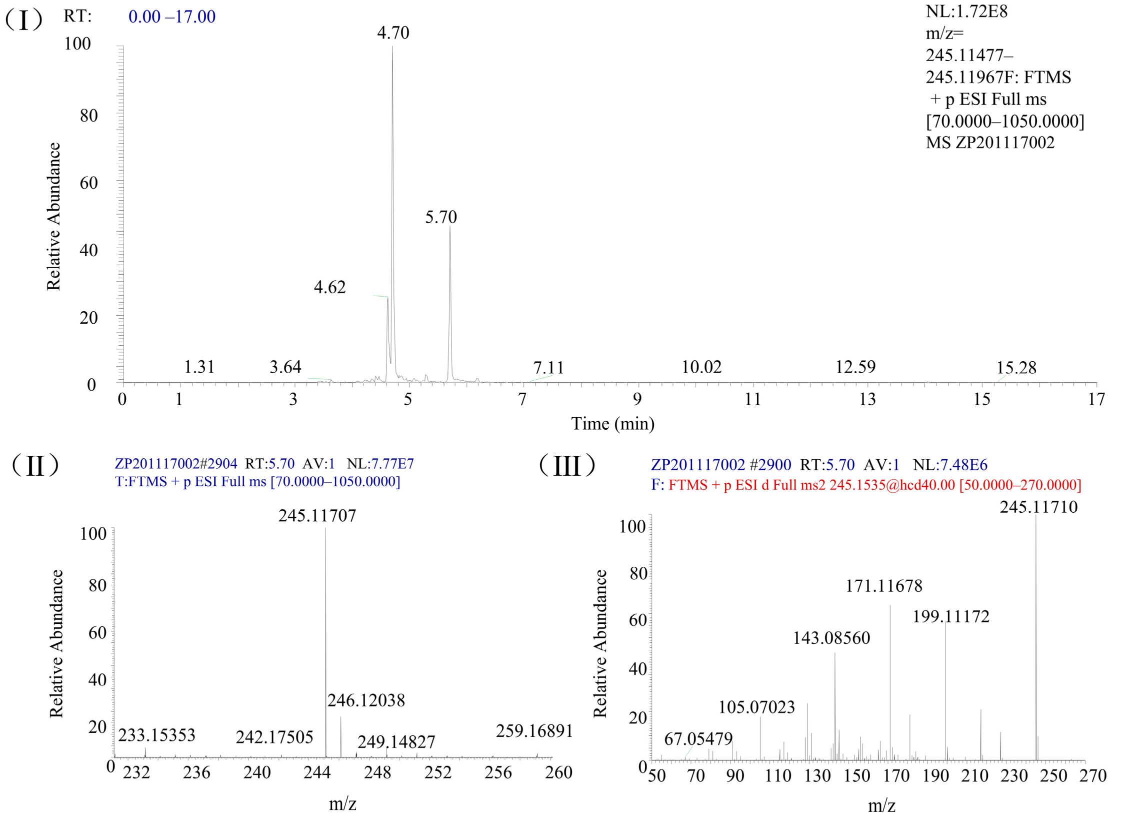

K

Artemisinic acid

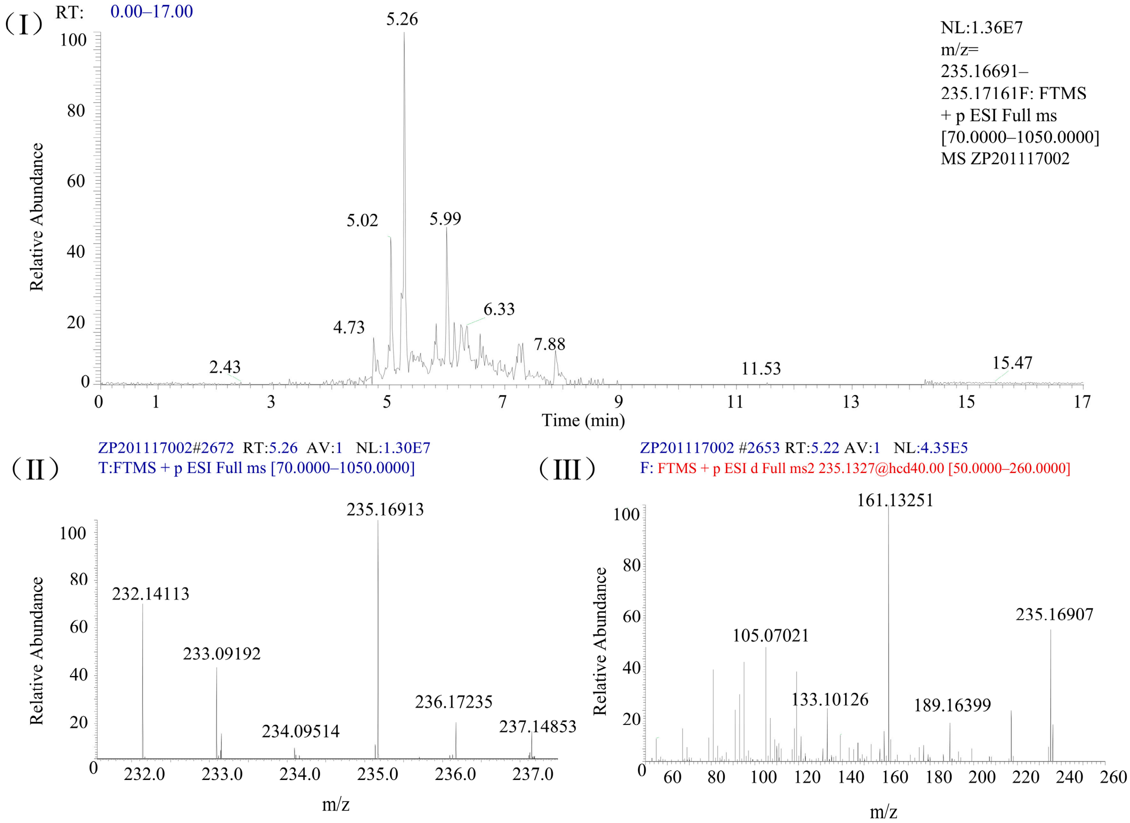

L

Isoalantolactone

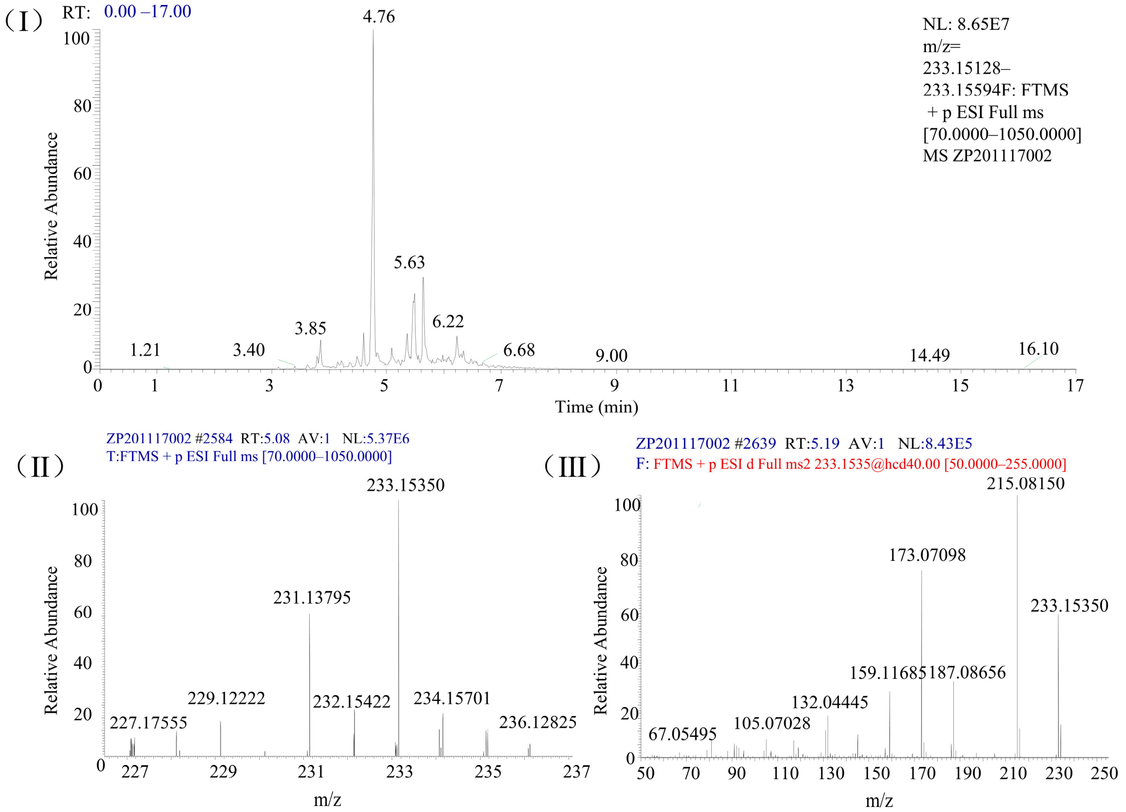

Figure S1. Untargeted qualitative analysis and identification of herbal chemical constituents based on LC-MS. **(A–L).** represent the mass spectrometric analysis results of betaine, (R)-mandelic acid, azelaic acid, arglabin, dehydrocostus lactone, arteannuin, nicotinic acid, atractylenolide II, parthenolide, linderalactone, artemisinic acid, and isoalantolactone, respectively. **I**: Full scan MS; **II**: MS1 spectra; **III**: MS2 spectra.

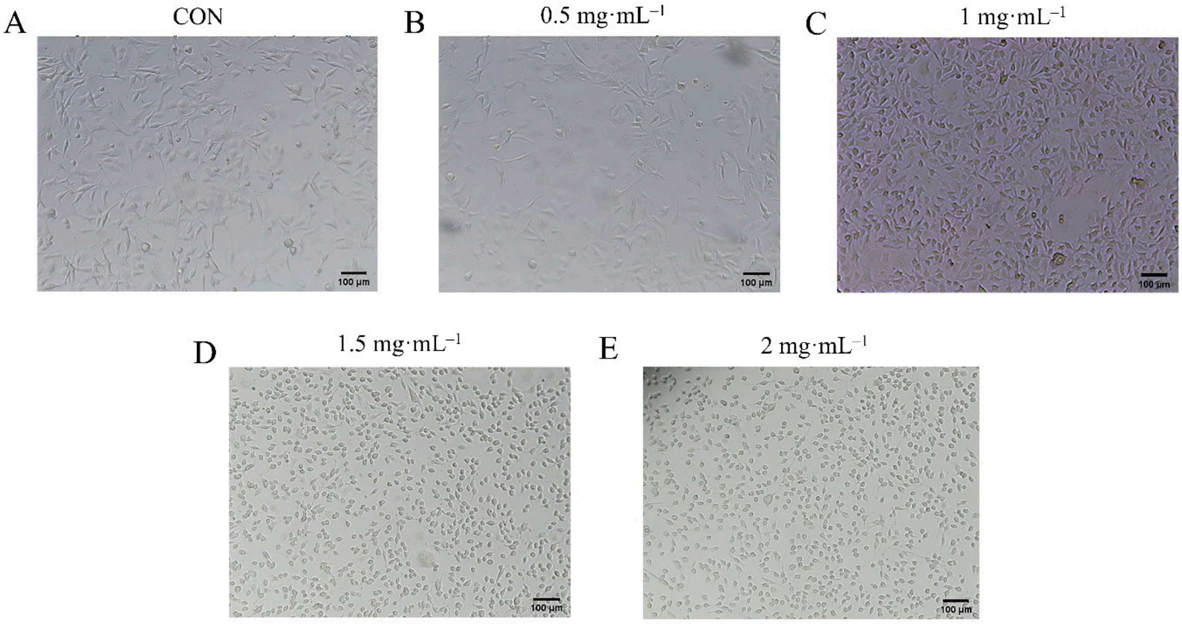

Figure S2. EA-2-induced growth inhibition and morphological changes in MDA-MB-231 breast cancer cells. **(A).** Control group. (**B–E).** EA-2 treatment groups at different concentrations. Scale bar 100 μm

S1 Label-Free Proteomics Analysis

S1.1 Mass Spectrometry Experimental Methods

S1.1.1 Protein Extraction and Digestion

Protein extraction was performed using SDT buffer (4% SDS, 100 mM Tris-HCl, 1 mM DTT, pH 7.6), where SDT refers to the combination of sodium dodecyl sulfate (SDS), dithiothreitol (DTT), and Tris-HCl. The protein concentration was determined with a

Bicinchoninic Acid Assay (BCA) Protein Assay Kit (Bio-Rad, USA). Protein digestion was carried out using the filter-aided sample preparation (FASP) method. Specifically, 200 μg of protein was mixed with 30 μL of SDT buffer, and detergents and small molecules were removed by ultrafiltration using 10 kDa filters. Cysteine residues were then alkylated with 100 mM iodoacetamide (IAA), followed by overnight digestion with 4 μg of trypsin at 37 °C. The resulting peptides were desalted using C18 columns, vacuum-dried, and reconstituted in 40 μL of 0.1% formic acid. Peptide concentration was determined by measuring UV absorbance at 280 nm.

S1.1.2 LC-MS/MS Analysis

Peptides were separated using an Easy nLC system (Thermo Fisher Scientific) and analyzed on a Q Exactive mass spectrometer (Thermo Scientific). Samples were first loaded onto a reverse-phase trap column (Thermo Scientific Acclaim PepMap100, 100 μm × 2 cm, C18) and then separated on an analytical C18 column (Thermo Scientific Easy Column, 10 cm × 75 μm, 3 μm) at a flow rate of 300 nL/min. The mobile phase consisted of buffer A (0.1% formic acid) and buffer B (84% acetonitrile, 0.1% formic acid). The mass spectrometer was operated in positive ion mode using a data-dependent acquisition (DDA) method, where the top 10 precursor ions were selected for higher-energy collisional dissociation (HCD) fragmentation. The MS scan range was set from 300 to 1800 m/z, with a resolution of 70,000 for MS and 17,500 for MS/MS.

S1.1.3 Protein Identification and Quantification

Raw MS data were analyzed using MaxQuant software (version 1.5.3.17) for protein identification and label-free quantification. Detailed parameters and explanations are

provided below:

Table S1 MaxQuant Identification and Quantification Parameters

Item	Value
Enzyme	Trypsin
Max Missed Cleavages	2
Main search	6ppm
First search	20ppm
MS/MS Tolerance	20ppm
Fixed modifications	Carbamidomethyl (C)
Variable modifications	Oxidation(M)
Database pattern	Reverse
Include contaminants	True
protein FDR	≤0.01
Peptide FDR	≤0.01
Peptides used for protein quantification	Use razor and unique peptides (Protein quantification was performed based on the intensity values of unique and razor peptides)
Time window (match between runs)	2min
protein quantification	LFQ
min. ratio count	1

S1.2 Bioinformatics Analysis

S1.2.1 Protein Clustering Analysis

The quantitative data of the target protein set were first normalized (scaled to the range of –1 to 1). Hierarchical clustering was then performed on both samples and protein expression levels using the ComplexHeatmap package in R (R version 3.4), employing Euclidean distance and average linkage as the clustering method. A hierarchical clustering heatmap was generated accordingly.

S1.2.2 Volcano Plot

To visualize the significance of differentially expressed proteins between comparison groups, volcano plots were drawn based on fold change (FC) and p-values (T-test). Proteins significantly downregulated ($FC < 0.5$ and $p < 0.05$) were marked in blue, significantly

upregulated proteins (FC > 2 and $p < 0.05$) in red, and non-significant proteins in gray.

S1.2.3 GO Functional Annotation

Gene Ontology (GO) annotation of the target protein set was performed using Blast2GO. The annotation process included four main steps: sequence alignment (Blast), GO term mapping (Mapping), GO annotation (Annotation), and annotation augmentation using InterProScan.

S1.2.4 KEGG Pathway Annotation

KEGG pathway annotation of the target protein set was conducted using the KEGG Automatic Annotation Server (KAAS).

S1.2.5 Enrichment Analysis of GO and KEGG Annotations

Fisher's exact test was used to compare the distribution of GO terms or KEGG pathways between the target and total protein sets. Enrichment analysis was then performed to identify significantly overrepresented GO terms or KEGG pathways in the target protein set.
